# Evaluation of the gut microbiome in association with biological signatures of inflammation in murine polytrauma and shock

**DOI:** 10.1038/s41598-021-85897-w

**Published:** 2021-03-23

**Authors:** Sandra A. Appiah, Christine L. Foxx, Dominik Langgartner, Annette Palmer, Cristian A. Zambrano, Sonja Braumüller, Evan J. Schaefer, Ulrich Wachter, Brooke L. Elam, Peter Radermacher, Christopher E. Stamper, Jared D. Heinze, Stephanie N. Salazar, Amalia K. Luthens, Andrea L. Arnold, Stefan O. Reber, Markus Huber-Lang, Christopher A. Lowry, Rebecca Halbgebauer

**Affiliations:** 1 Department of Integrative Physiology and Center for Microbial Exploration, University of Colorado Boulder, Boulder, CO 80309 USA; 2 Laboratory for Molecular Psychosomatics, Department of Psychosomatic Medicine and Psychotherapy, University Ulm, 89081 Ulm, Germany; 3 Institute of Clinical and Experimental Trauma Immunology, Centre for Biomedical Research, University Hospital Ulm, University Ulm, Helmholtzstr. 8/1, 89081 Ulm, Germany; 4 Center for Neuroscience, University of Colorado Boulder, Boulder, CO 80309 USA; 5 Institute for Anaesthesiological Pathophysiology and Process Development, University of Ulm, Ulm, Germany; 6 Department of Physical Medicine and Rehabilitation and Center for Neuroscience, University of Colorado Anschutz, Medical Campus, Aurora, CO 80045 USA; 7 Rocky Mountain Mental Illness Research Education and Clinical Center (MIRECC), Rocky Mountain Regional Veterans Affairs Medical Center (RMRVAMC), Aurora, CO 80045 USA; 8 Military and Veteran Microbiome Consortium for Research and Education (MVM-CoRE), Aurora, CO 80045 USA

**Keywords:** Immunology, Microbiology

## Abstract

Severe injuries are frequently accompanied by hemorrhagic shock and harbor an increased risk for complications. Local or systemic inflammation after trauma/hemorrhage may lead to a leaky intestinal epithelial barrier and subsequent translocation of gut microbiota, potentially worsening outcomes. To evaluate the extent with which trauma affects the gut microbiota composition, we performed a post hoc analysis of a murine model of polytrauma and hemorrhage. Four hours after injury, organs and plasma samples were collected, and the diversity and composition of the cecal microbiome were evaluated using 16S rRNA gene sequencing. Although cecal microbial alpha diversity and microbial community composition were not found to be different between experimental groups, norepinephrine support in shock animals resulted in increased alpha diversity, as indicated by higher numbers of distinct microbial features. We observed that the concentrations of proinflammatory mediators in plasma and intestinal tissue were associated with measures of microbial alpha and beta diversity and the presence of specific microbial drivers of inflammation, suggesting that the composition of the gut microbiome at the time of trauma, or shortly after trauma exposure, may play an important role in determining physiological outcomes. In conclusion, we found associations between measures of gut microbial alpha and beta diversity and the severity of systemic and local gut inflammation. Furthermore, our data suggest that four hours following injury is too early for development of global changes in the alpha diversity or community composition of the intestinal microbiome. Future investigations with increased temporal-spatial resolution are needed in order to fully elucidate the effects of trauma and shock on the gut microbiome, biological signatures of inflammation, and proximal and distal outcomes.

## Introduction

Polytrauma (PT) as life-threatening multiple injuries is the most frequent cause of death for people under the age of 45. Hemorrhagic shock (HS) frequently accompanies severe tissue injuries observed in PT and the additive effects of HS on PT remain associated with high morbidity and mortality rates despite modern intensive care and surgical damage control strategies^1^. Clinical cohort studies note that PT frequently affects the brain, lungs, and endothelium^2–4^. We have recently established a mouse model of severe multiple injuries and blood loss corresponding to severe PT + HS^5^. PT was induced by a combination of closed blunt traumatic brain injury (TBI), blunt thorax trauma (TXT), soft tissue injury, and femur fracture^5^. Compared to PT alone, a combination of PT with an additional severe HS further negatively impacts the lungs by inducing histological damage, pulmonary edema, and neutrophil influx. Furthermore, HS following PT induces systemic increases in proinflammatory cytokine concentrations and increases the demand for administration of norepinephrine (NE) support to restore blood pressure^5^. Moreover, PT + HS negatively impacts renal function, induces intestinal damage, and reduces the intestinal expression of tight junction protein zonula occludens protein-1 (ZO-1), even in the absence of any direct trauma to either the kidneys or intestines^6^.

While the exact mechanisms underlying the variable characteristics of trauma-related complications are still unexplained, studies suggest that hyperreactive local and systemic immune responses promote post-trauma risk for complications. For instance, studies combining TXT with a cecal ligation/puncture-induced sepsis model show that severe local and systemic inflammatory responses substantially promote the development of acute lung injury (ALI) following TXT in mice^7^. In line with these findings, human studies indicate that the development of multiple organ failure (MOF) and subsequent mortality is associated with exaggerated local or systemic inflammation at an early time point following trauma^8,9^. Similarly, HS-mediated hyper-inflammation precedes negative post-trauma clinical outcomes including sepsis and MOF^10^.

Together, these findings support the hypothesis that factors contributing to an exaggerated local and systemic immune activation following PT increase an individual’s complication risk. Genetic predisposition^11^, aging^12^, and pre-existing co-morbidities^13–15^ influence local gut and systemic immune responses; however, it is becoming clear that other factors also influence local gut and systemic immune responses, including epithelial barrier function and the composition of the gut microbiome. For example, reports of epithelial barrier defects^16^ and colitogenic gut dysbiosis following chronic psychosocial trauma^17^, physical trauma, or a combination of both^18^, are accompanied by increased local colonic immune activation^19^ and systemic immune activation^18,20^.

In the context of physical trauma, several studies demonstrate that different forms of injury affect the composition of the gut microbiome within the first 72 h post injury^21,22^ and that alterations of the taxonomic composition of the intestinal microbiome correlate with the severity of critical illness following trauma^23^. In line with these findings, gastrointestinal dysbiosis triggered by trauma or surgical intervention is further associated with the severity of systemic inflammation^24^. Intestinal dysfunction is a common characteristic of TBI^25^, and HS has been implicated as an engine for increased morbidity and mortality in PT patients, with gut-barrier dysfunction as a key hallmark^26^.

Given these findings, it is likely that changes in the composition of the gut microbiome are also part of the pathophysiological mechanisms underlying the local and systemic immune activation seen in our experimental model of PT + HS. Therefore, we hypothesize that there are measurable alterations in the composition of the intestinal microbiome of PT + HS mice as early as 4 h after injury that, together with the already described increase in gut permeability markers in these mice, act in concert to promote local and systemic inflammation^5,6^. To test this hypothesis, we analyzed the microbial composition of samples of cecal contents, taken from animals of our previously published studies^5,6^, employing 16 small subunit ribosomal RNA (16S rRNA) gene sequencing, and correlated the diversity and community structure of the microbiome with the individual immunological parameters assessed in each mouse.

## Materials and methods

### Animals

This study is a subanalysis of a previously published murine study of polytrauma and hemorrhage^5,6^. Briefly, male C57BL/6 J mice (*N* = 36; Jackson Laboratories, Bar Harbor, Maine, USA) with a mean body weight of 25 g (± 2.5 g) were ordered from the animal vendor over a study period of eight months (August 2015 until March 2016) in batches to ensure equal age at the beginning of experimental procedures. Mice were acclimated to the local animal facility under the following identical housing conditions: co-housing with 5 mice/cage, light/dark cycle of 14/10 h (lights on at 6 a.m. CET), 22 °C, and 60% humidity. Animals had free access to food pellets (V1535-000, composition: 22% crude protein, 4.5% crude fat, 3.9% crude fiber, 6.7% crude ash, 1% calcium, 0.7% phosphorus; ssniff Spezialdiäten GmbH, Soest, Germany) and non-acidified autoclaved tap water. Additionally, Lignocel/Premium Scientific Bedding (Rettenmaier & Söhne, Rosenberg, Germany) was used as litter. The study protocol was approved by the University Animal Care Committee and the Federal Authorities for animal research, Tuebingen, Germany (No. 1194). Moreover, all experiments were performed in adherence to the National Institutes of Health *Guide for the Care and Use of Laboratory Animals, 8*^*th*^* edition* (2011) and were consistent with the ARRIVE 2.0 Guidelines.

### Induction of PT and HS

**Figure 1:**
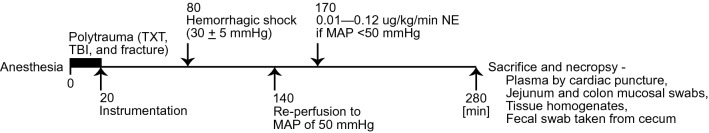
The experimental timeline for the murine polytrauma (PT) with hemorrhagic shock (HS) model. All mice were randomly subjected to PT, HS, PT + HS, or sham-catherization procedures (*N* = 36; *n* = 8 per group, PT, HS, Sham; *n* = 7 per group, PT + HS). Untreated control mice (Ctrl, *n* = 5) are not shown here. Briefly, all mice were anaesthetized with 2.5–3% sevoflurane in oxygen. Ctrl animals were euthanized directly after induction of anesthesia. Mice in the PT and PT + HS groups were subjected to thoracic trauma/blunt bilateral chest trauma (TXT), closed head injury (TBI), and a distal femur fracture accompanied by soft tissue injury. At 20 min, mice in the Sham, HS, and PT + HS groups were catheterized in the left femoral artery to monitor blood pressure and induce sham procedures or HS; a jugular vein catheter was also inserted for reperfusion following blood loss. At the 80 min time point, mice in the HS and PT + HS groups were bled for 5–10 min to reach a mean arterial pressure (MAP) of 30 ± 5 mmHg which was kept stable for 60 min. At 140 min, mice in the HS and PT + HS groups were reperfused through the jugular vein with a balanced electrolyte solution over 30 min, using a fourfold volume calculation of the blood drawn to induce HS. Mice that failed to recover a MAP of 50 mmHg were additionally supported by a predetermined anesthesia adjustment and norepinephrine support protocol, by which the goal MAP of 50 mmHg was attained. *MAP* mean arterial pressure, *NE* norepinephrine, *PT* polytrauma, *TBI* traumatic brain injury, *TXT* thoracic trauma/blunt bilateral chest trauma.

The experimental timeline is illustrated in Fig. 1 and was performed as described previously^5,6^. After at least 10 days of acclimation at the animal facility, mice were randomly assigned to 5 different experimental groups (N = 36: polytrauma (PT, *n* = 8), hemorrhagic shock (HS, *n* = 8), polytrauma + hemorrhagic shock (PT + HS, *n* = 7), anaesthetized and catheterized-sham controls (Sham, *n* = 8) and untreated controls (Ctrl, *n* = 5). Mice were anaesthetized with 2.5–3% sevoflurane (Sevorane Abbott, Wiesbaden, Germany) in oxygen, which was continued during the trauma-hemorrhagic procedure and during the whole observation period. For analgesia, 0.03 mg/kg buprenorphine (Temgesic, RB Pharmaceutics, Slough, UK) was administered by subcutaneous injection 30 min before initiation of trauma. Untreated control animals were euthanized directly after induction of anesthesia by cardiac puncture. The PT procedure was performed as described elsewhere^5,6^. Briefly, PT was induced by application of a blunt bilateral chest trauma (TXT), closed head injury (TBI), and a distal femur fracture accompanied by soft tissue injury.

Following PT induction, the left hind limb was shaved and disinfected, an incision was made, and a catheter (Föhr Medical Instruments, Seeheim/Ober-Beerbach, Germany) was micro-surgically inserted into the femoral artery. This allowed the monitoring of blood pressure by a blood pressure analyzer (Data Sciences International, DSI, St. Paul, Minnesota, USA) and a pressure-controlled blood loss to induce HS. Another incision was made along the ventral cervical skin through which a catheter was inserted into the jugular vein, which enabled us to resuscitate animals (see below) and control the infusion of NE. The full HS procedure was also performed as described elsewhere^5^. Briefly, mice were bled for 5–10 min to reach a mean blood pressure of 30 mmHg (± 5 mmHg) which was kept stable for 60 min. After hemorrhage, mice were resuscitated with a sterile balanced electrolyte solution (Jonosteril, Servoprax, Wesel, Germany) via the jugular vein using fourfold the volume of drawn blood over 30 min. After termination of this procedure, animals underwent a 2 h observation period. During the 2 h observation period, animals were subjected to a preset protocol, adjusting anesthesia and catecholamine (i.e., NE) support (0.01–0.12 µg/kg/min; Sanofi, Frankfurt am Main, Germany) in a standardized manner to maintain a mean arterial pressure (MAP) of ≥ 50 mmHg. The amount of transfused fluid, NE received, as well as blood hemoglobin (Hb) concentrations, were documented for each animal.

### Plasma and intestinal endpoints

Four hours after PT, blood was drawn by cardiac puncture into ethylenediaminetetraacetic acid (EDTA)-coated tubes. Plasma was obtained by centrifugation (800 × *g* and 4 °C for 5 min; supernatants were centrifuged again at 13,000 × *g* and 4 °C for 2 min) and stored at – 80 °C until cytokines and proteins of interest could be measured via enzyme-linked immunosorbent assay (ELISA). Cytokines and proteins of interest that were assessed included interleukin 6 (IL-6), junctional adhesion molecule A (JAM-A), mucin-2, and monocyte chemoattractant protein-1 (MCP-1). Following final blood draw, additional tissue samples were taken during necropsy for further analysis. Mucus was removed from the jejunum and colon, and tissue homogenates were used for the assessment of complement component C3a (C3a), complement component C5a (C5a), and IL-6; jejunum and colon tissue occludin were also quantified. Finally, the cecum was opened aseptically to sample cecal contents for microbial analysis. Cecal samples were stored in sterile tubes at – 80 °C until further analysis. Some readouts assessed in these mice (plasma IL-6 concentrations and creatinine concentrations normalized to total plasma protein) have been previously reported^5^.

### Homogenization of jejunum and colon tissue

Approximately 10 cm of the jejunum and the entire colon were extracted, flushed with 10 ml ice-cold phosphate-buffered saline (PBS) each, and dissected longitudinally. Mucus was removed using a glass cover slip. The jejunum and colon tissues were homogenized using a mechanical disperser (17,500 rpm, IKA, Staufen, Germany) and sonicated (5 cycles of 5 s each, Bandelin electronics, Berlin, Germany) in PBS containing a 1:1000 broad spectrum protease inhibitor (Sigma-Aldrich, St. Louis, Missouri, USA). After centrifugation (15 min, at 16,000 × *g* and 4 °C), the supernatant was collected and stored at – 80 °C until further analysis.

### Enzyme-linked immunosorbent assay (ELISA)

ELISAs and sequential ELISA measurements were performed as described previously^5,6,27^. The following ELISA kits were used according to the manufacturer’s recommendations: BD OptEIA Mouse IL-6 ELISA Set and BD OptEIA Mouse MCP-1 ELISA Set (both BD Pharmingen, San Diego, CA, USA), Mouse JAM-A DuoSet ELISA and Mouse Complement Component C5a DuoSet ELISA (both R&D Systems, Minneapolis, MN, USA), Mouse Occludin ELISA Set (Wuxi Donglin Sci&Tech Development, Jiangsu, P.R.C.), and Mouse Mucin 2 / MUC2 (Sandwich ELISA) ELISA Kit (LSBio, Seattle, WA, USA). Activated complement component C3a was detected using an in-house sandwich ELISA employing antibodies purchased from BD Pharmingen and a protein standard purchased from R&D Systems. The measured concentrations of parameters in the jejunum and colon tissue homogenates were calculated relative to the total protein concentration in the corresponding sample.

### Quantification of plasma creatinine

Plasma creatinine concentrations were measured as described in Denk et al.^5^. Briefly, mouse plasma samples were mixed with internal standard solution, deproteinized and centrifuged at 13,000 × *g* for up to 5 min; supernatants were used for urea quantification on an Agilent 5890/5970 gas chromatography/ mass spectrometry system (Macherey–Nagel, Düren, Germany). Six-point calibration curves were used for quantification^28^.

### Bacterial DNA extraction and generation of 16S rRNA gene V4 amplicons

Bacterial genomic DNA extraction, hypervariable region 4 (V4) amplicon generation from the 16S rRNA gene, and amplicon preparation for sequences were performed as described previously^29^ and according to protocols benchmarked for the Earth Microbiome Project (EMP) which can be found on the EMP website (http://www.earthmicrobiome.org/emp-standard-protocols/). Bacterial genomic DNA was extracted from samples using the DNeasy PowerSoil 96-well DNA isolation kit (Cat. no. 12955-4, Qiagen Laboratories, Hilden, Germany) according to manufacturer’s instructions. Marker genes in isolated DNA were PCR-amplified in duplicate from each sample, targeting V4 of the 16S rRNA gene, modified with a unique 12-bp sequence identifier for each sample and the Illumina adapter, as previously described by Caporaso et al.^30^.

PCR was performed as described elsewhere^29^. The PCR mixtures contained 13 µl Mo Bio PCR water, 10 µl 5′-HotMasterMix, 0.5 µl each of the barcoded forward and reverse primers (515-bp forward: 5′-GTGCCAGCMGCCGCGGTAA-3′; 806-bp reverse: 5′-GGACTACHVGGGTWTCTAAT-3′; 10 µM final concentration, Integrated DNA Technologies, San Diego, CA, USA), and 1.0 µl genomic DNA. Reaction mixtures were held at 94 °C for 3 min, followed by 35 cycles of amplification (94 °C for 45 s, 50 °C for 1 min, and 72 °C for 1.5 min), followed by a final extension at 72 °C for 10 min. PCR products were cleaned and normalized using the SequalPrep Normalization Kit (Cat. No. A1051001, ThermoFisher, Waltham, MA, USA).

### 16S rRNA gene sequence and data preparation

Normalized amplicons were sequenced at the BioFrontiers Next Generation Sequencing Core Facility at the University of Colorado Boulder using the Illumina MiSeq platform. The 16S rRNA gene library concentration was measured using the HiSens Qubit dsDNA HS assay kit (Cat. No. Q32854, ThermoFisher Scientific, Waltham, MA, USA). A total of 6 pM of the 16S rRNA gene library combined with 0.9 pM (15%) PhiX sequencing library control v3 (Cat. no. FC-110, 3001, Illumina Inc., San Diego, CA, USA) was sequenced with 150-bp paired-end reads on an Illumina MiSeq sequencing system using a MiSeq reagent kit v3 (2 × 300 cycles; Cat. no. MS-102–2002, Illumina Inc.). FASTQ files for reads 1 (forward), 2 (reverse), and the index (barcode) read were generated using the BCL-to-FASTQ file converter bcl2fastq (ver. 2.17.1.14, Illumina, Inc.).

Sequencing data were primarily prepared and analyzed using the Quantitative Insights Into Microbial Ecology microbiome analysis pipeline (QIIME2 ver. 2020.2, http://qiime2.org; Bolyen et al.^31^). Mapping files and raw sequencing information are publicly available on the microbiome study management platform Qiita (http://qiita.ucsd.edu/study/description/12753; Gonzalez et al.^32^). Sequence reads were filtered employing the Divisive Amplicon Denoising Algorithm (DADA2) default parameters as described previously^33,34^: expected error threshold of 2, trimming of 19 nucleotides from the start of the forward reads to remove the 515f.’ primer, and trimming of 20 nucleotides from the start of the reverse reads to remove the 806r’ primer^35^. Filtered reads were then de-replicated and de-noised using DADA2 default parameters to combine identical reads into unique amplicon sequence variants (ASVs) and construct consensus quality profiles for each combined lot of sequences; the consensus quality profiles then inform the de-noising algorithm, which infers error rates from samples and removes identified sequencing errors from the samples as described by Dahan et al.^33^. Following processing of raw sequence reads through the DADA2 pipeline, the data were constructed into a feature table of 426 unique ASVs with an average read length of 237.28 ± 2.28 nucleotides in 36 samples.

After building the feature table and removing chimeras, phylogenetic trees were built using the SaTé-enabled phylogenetic placement (SEPP) fragment-insertion classifier implemented in QIIME2 using the q2-fragment-insertion plugin^36,37^, trained against the SILVA_128 99% ribosomal gene reference database built on the 16S rRNA gene V4 region using the same primers as above^30,38^. Lastly, we filtered outgroups not present in the SEPP insertion tree from the ASV table prior to rarefaction and calculation of phylogenetic diversity metrics such as Faith’s phylogenetic diversity^39^ or UniFrac^40^.

### Diversity and differential data analysis of 16S rRNA gene sequence data

Microbial community structure was characterized using measures of alpha diversity (within-sample diversity) and beta diversity (between-samples dissimilarity). Metrics of alpha diversity included, but were not limited to, the number of distinct features to represent species richness, Pielou’s *J’* to represent species evenness^41^, Shannon’s diversity index H’ to represent species abundance and evenness^42^, and Faith’s phylogenetic diversity, which measures the total length of branches in a reference phylogenetic tree for all species in a given sample^39^. Beta diversity was calculated using unweighted and weighted UniFrac distances^40,43,44^ depicting community-wide differences in microbial composition amongst cecal samples. Output distance matrices were ordinated using principal coordinate analyses (PCoA) and visualized using EMPeror^45^. For a glossary of microbial ecology and microbiome analysis terms used, please refer to Table 1.

**Table 1:** Glossary of microbial analysis terms.

Term	Definition
Phylogeny	The evolution of a group of organisms relative to a common ancestor based on genetics, represented as a tree diagram with nodes (points of genetic divergence or speciation), branches (descendants of a common ancestor), and tips (individual species)
Taxonomy	The classification of a group of organisms relative to other groups or individual organisms based on shared characteristics (e.g., target sequence similarity)
Richness	The total number of organisms in a sample measured as a count of unique sequences (e.g., number of distinct features)
Evenness	The relative distribution of organisms in a sample measured as a function of unique sequences and counts of each unique sequence (e.g., Pielou’s evenness index $J$’)
Alpha-diversity	Variance within a single sample described as a metric of either species richness, evenness, or both (diversity, e.g. Shannon’s diversity index $H$’)
Beta-diversity	Variance in communities between multiple samples represented as a matrix of scores that quantify each individual sample’s similarity or dissimilarity when compared to all other samples in a population of interest

We used Analysis of Composition of Microbiomes (ANCOM)^46^ to identify taxa driving differences in microbial composition between treatment groups over time. Prior to conducting ANCOM, we pre-processed the ASV table to remove structural zeros from the data matrix and impute sampling zeros with a pseudo-count of 1 as described in the ANCOM-II methods^47^. No outlier zeros were identified in R as described in Kaul et al.^47^, so NA values were not substituted for any zero feature counts in the ASV table.

Additionally, we utilized a supervised machine-learning algorithm to predictively classify individual samples as having low or high inflammatory marker levels based on microbial features. Using a nested cross-validated (*k* = fivefold) strategy, the receiver-operating characteristic (ROC) area-under-the-curve (AUC) values for the ASV-based models that were used to calculate feature importances varied between 0.65 and 0.70. All analyzed data are provided in Supplemental File 1.

### Statistical approach

Statistical analysis in QIIME2 of each alpha diversity metric described above, using Kruskal–Wallis tests and post hoc pairwise Mann–Whitney U tests without adjustment for multiple testing, were conducted on all categorical variables in the metadata describing each sample submitted for 16S rRNA gene sequencing. These variables included treatment group, trauma status, and transfused fluid in tertiles, and the following variables split into halves: NE used for blood pressure maintenance (*N* = 11; *n* = 7, high NE; *n* = 4, low NE), biological signatures of inflammation in tissue homogenates of the colon and jejunum (C3a, C5a, IL-6, and occludin), and biological signatures of inflammation, barrier breakdown, and organ dysfunction in plasma drawn during tissue collection as described previously (C3a, creatinine, IL-6, JAM-A, MCP-1, and mucin-2).

Additional analyses of each alpha diversity metric described above, using Pearson’s correlations in QIIME2, were conducted on the rarefied feature table at a two-tailed alpha threshold of 0.05 against all continuous variables in the metadata describing each sample submitted for 16S rRNA gene sequencing. Statistical significance of beta-diversity distances between groups described in the categorical variables above were assessed using permutational analysis of variance (PERMANOVA) with 999 Monte Carlo permutations and post hoc pairwise permutational *t*-tests^48^.

To identify taxa driving differences in microbial composition between treatment groups, the pre-processed ASV table was then used in a linear mixed-effects model of experimental day × treatment condition using the ANCOM-II implementation in R (source code: http://github.com/FrederickHuangLin/ANCOM). We chose a W-statistic cutoff value of 0.8 where the *p*-value obtained for each taxon was corrected for multiple testing against all other taxa using a Benjamini–Hochberg false discovery rate (FDR) adjustment with significant taxonomic abundance differences reported at an alpha-threshold of 0.05.

## Results

### Alpha-diversity analysis

We first analyzed whether there were significant alterations in measures of microbial alpha-diversity depending on group allocation of the animals. However, throughout all parameters assessed, comparisons of the varying treatment groups showed no significant differences in the between-groups alpha-diversity analysis of the cecal microbiome (data not shown). We then proceeded to separate animals according to experimental and biological variables in those with low and those with high values, and assessed differences in alpha-diversity metrics.

**Figure 2:**
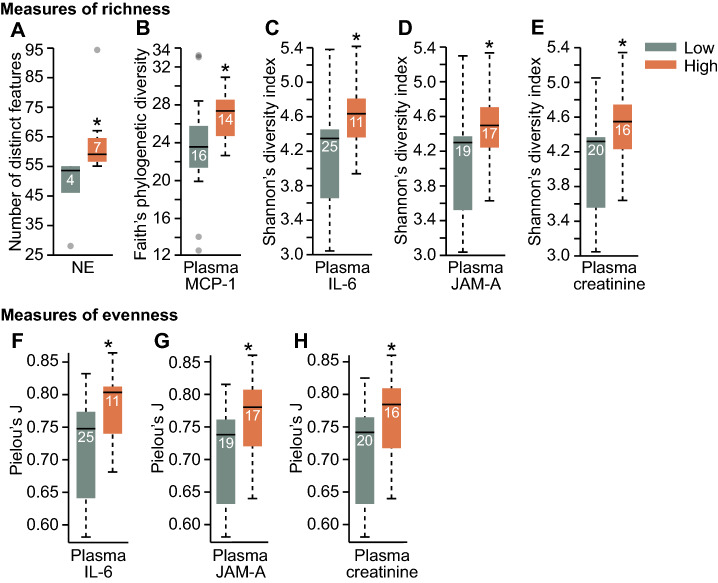
Maintenance of blood pressure, as measured by total norepinephrine (NE) administered, and several plasma measures of inflammation and membrane damage are associated with elevated levels of microbial alpha diversity. **(A)** Number of distinct features, a metric of richness, against low or high total NE (µg) required for maintenance of an appropriate blood pressure following polytrauma (PT) + hemorrhagic shock (HS), or HS alone, **(B)** Faith’s phylogenetic diversity against low or high plasma monocyte chemoattractant protein-1 concentration (MCP-1, pg/ml), **(C)** Shannon’s diversity index against low or high plasma interleukin-6 concentration (IL-6, pg/ml), **(D)** Shannon’s diversity index against low or high plasma junctional adhesion molecule A concentration (JAM-A, pg/ml), **(E)** Shannon’s diversity index against low or high plasma creatinine concentration (pg/ml), **(F–H)** Pielou’s *J’* against **(F)** low or high plasma IL-6 concentration (pg/ml), **(G)** low or high plasma JAM-A concentration (pg/ml), and **(H)** low or high plasma creatinine concentration (pg/ml). The bottoms and tops of boxes indicate the first and third quartiles, respectively; whiskers indicate the 1.5 interquartile range beyond the upper and lower quartiles. Values outside the whiskers are indicated by gray circles; numbers in each box indicate sample sizes. Statistical significance in each categorical variable was evaluated using Kruskal–Wallis ranks-sums tests with post hoc pairwise Mann–Whitney *U* tests at *p* < 0.05. *p < 0.05; *HS* hemorrhagic shock, *IL-6* interleukin 6, *IQR* interquartile range, *JAM-A* junctional adhesion molecule A, *MCP-1* monocyte chemoattractant protein-1, *NE* norepinephrine, *PT* polytrauma.

Mice that had undergone HS with or without PT (*N* = 11; high NE, *n* = 3 HS and *n* = 4 PT + HS; low NE, *n* = 3 HS and *n* = 1 PT + HS) required administration of high doses of NE (> 0.04 µg) for blood pressure maintenance. As previous studies have shown that NE acts directly on some strains of bacteria to alter chemotaxis and/or increase proliferation^49–52^, we compared measures of alpha diversity in mice receiving high versus low doses of i.v. NE; mice that did not require any NE support for blood pressure maintenance (*n* = 25) were excluded from this assessment. Non-parametric analyses demonstrated that animals requiring high levels of NE for blood pressure maintenance had a higher median number of distinct features compared to low NE animals (*H*-statistic = 6.15, *p* = 0.013; Fig. 2A). When we expanded our alpha-diversity analysis to other metrics of richness (i.e., Faith’s phylogenetic diversity and Shannon’s diversity index), we generally found that high microbial richness was associated with high levels of plasma cytokines and membrane proteins (Fig. 2B-E). For example, Faith’s phylogenetic diversity was positively associated with high plasma MCP-1 (*N* = 30; six samples were not analyzed due to limited plasma available for multiple analytes; *H*-statistic = 4.15, *p* = 0.042; Fig. 2B). Among all treatment groups, we found that a disproportionate number of mice in the high plasma MCP-1 group had received PT + HS (low plasma MCP-1, *n* = 6 HS, *n* = 3 PT, *n* = 2 PT + HS, *n* = 4 Sham, *n* = 1 Ctrl; high plasma MCP-1, *n* = 2 HS, *n* = 3 PT, *n* = 5 PT + HS, *n* = 4 Sham).

Shannon’s diversity, which has been shown to represent alpha diversity as a mixture of both evenness and richness^53^, was positively associated with plasma IL-6 concentrations (*N* = 36; *H*-statistic = 4.18, *p* = 0.041; Fig. 2C). We showed here, as in previous findings from this dataset, that elevated IL-6 is positively associated with damage severity^5,6^, with a predominant increase of PT + HS representation within the high IL-6 group (low plasma IL-6, *n* = 7 HS, *n* = 3 PT, *n* = 2 PT + HS, *n* = 8 Sham, *n* = 5 Ctrl; high plasma IL-6, *n* = 1 HS, *n* = 5 PT, *n* = 5 PT + HS). Similarly, high plasma JAM-A (*N* = 36; *H*-statistic = 4.44, *p* = 0.035; Fig. 2D) and plasma creatinine (*N* = 36; *H*-statistic = 4.83, *p* = 0.028; Fig. 2E) were associated with increased alpha diversity. We found here, as in other alpha-diversity analyses of treatment groups, that PT + HS mice were predominantly in the high JAM-A group (low plasma JAM-A, *n* = 3 HS, *n* = 5 PT, *n* = 1 PT + HS, *n* = 6 Sham, *n* = 4 Ctrl; high plasma JAM-A, *n* = 5 HS, *n* = 3 PT, *n* = 6 PT + HS, *n* = 2 Sham, *n* = 1 Ctrl). Additional exploration of the data demonstrated that the same was true of high plasma creatinine (low plasma creatinine, *n* = 2 HS, *n* = 5 PT, *n* = 2 PT + HS, *n* = 6 Sham, *n* = 5 Ctrl; high plasma creatinine, *n* = 6 HS, *n* = 3 PT, *n* = 5 PT + HS, *n* = 2 Sham).

Further analysis of microbial alpha diversity in the context of evenness, as measured by Pielou’s *J’*, confirmed that systemic inflammation was associated with changes in microbial diversity. Kruskal–Wallis rank-sum analysis of plasma IL-6 concentrations demonstrated that high plasma IL-6 concentrations were associated with increased evenness (*H*-statistic = 4.04, *p* = 0.045; Fig. 2F). Similarly, we found consistent relationships in evenness as in prior analyses with Shannon’s diversity index with plasma JAM-A (*H*-statistic = 5.13, *p* = 0.023; Fig. 2G) and plasma creatinine (*H*-statistic = 4.28, *p* = 0.039; Fig. 2H).

**Figure 3:**
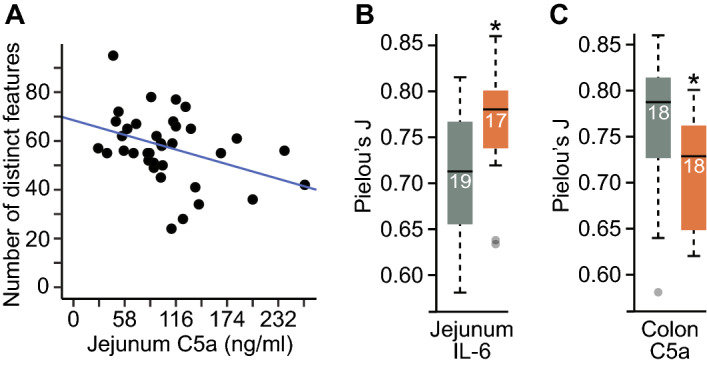
Alpha-diversity analysis with Pielou’s *J’*, a metric of evenness, against various cytokines and chemokines localized to the intestinal region using **(A)** Pearson’s correlations with jejunum complement component 5a concentration (C5a, ng/ml) against number of distinct features, and **(B)** Kruskal–Wallis rank-sum tests with post hoc pairwise Mann–Whitney *U* tests with jejunum interleukin-6 concentration (IL-6, pg/ml) and **(C)** colon C5a concentration (ng/ml) against Pielou’s *J’*. The bottoms and tops of boxes indicate the first and third quartiles, respectively; whiskers indicate the 1.5 interquartile range beyond the upper and lower quartiles. Values outside the whiskers are indicated by gray circles; numbers in each box indicate sample sizes. Statistical significance in each categorical variable was evaluated using Kruskal–Wallis rank-sum tests with post hoc pairwise Mann–Whitney *U* tests at *p* < 0.05. *p < 0.05. *C5a* complement component 5a, *IL-6* interleukin 6.

We also performed Pearson’s correlation analyses of all four alpha-diversity metrics against continuous measurements of Jonosteril or NE transfused for blood pressure maintenance, as well as measurements of cytokines, chemokines, and membrane integrity proteins. Only one statistically significant correlation was observed; number of distinct features, a measure of microbial richness, was inversely associated with jejunum C5a such that high jejunum C5a samples had low microbial diversity (*N* = 36; *r* = –0.37, *p* = 0.026; Fig. 3A). Investigation of the relationships between localized intestinal inflammation and microbial alpha diversity showed that high jejunum IL-6 concentrations were associated with high Pielou’s *J’* (*N* = 36; *H*-statistic = 5.28, *p* = 0.022; Fig. 3B) with a tendency for higher jejunum IL-6 in HS and HS + PT mice (low jejunum IL-6 concentration, *n* = 2 HS, *n* = 6 PT, *n* = 3 PT + HS, *n* = 3 Sham, *n* = 5 Ctrl; high jejunum IL-6 concentration, *n* = 6 HS, *n* = 2 PT, *n* = 4 PT + HS, *n* = 5 Sham). Colon C5a, a marker of acute intestinal inflammation induced by innate immune mechanisms, was inversely associated with Pielou’s *J’* such that high colon C5a concentrations were associated with low microbial evenness (*N* = 36; *H*-statistic = 5.19, *p* = 0.023; Fig. 3C). We did not find any associations between injury severity and colon C5a concentrations although C5a was tendentially higher in Sham and Ctrl animals (low colon C5a, *n* = 6 HS, *n* = 4 PT, *n* = 6 PT + HS, *n* = 2 Sham; high colon C5a, *n* = 2 HS, *n* = 4 PT, *n* = 1 PT + HS, *n* = 6 Sham, *n* = 5 Ctrl).

### Beta-diversity analysis to identify changes in microbial composition

As certain protein markers of inflammation and membrane damage may be associated with compositional changes in the microbiome, we conducted PERMANOVAs on the unweighted and weighted UniFrac distance matrices generated from the rarefied feature table. Analysis of weighted UniFrac in the comparisons of the varying treatment groups showed no significant differences in the between-groups beta-diversity analysis of the cecal microbiome (*pseudo-F* = 1.00, *p* = 0.463; data not shown). Analysis of unweighted UniFrac in the comparisons of the varying treatment groups showed no significant differences in the between-groups beta-diversity analysis of the cecal microbiome (*pseudo-F* = 0.58, *p* = 0.999; data not shown).

**Figure 4:**
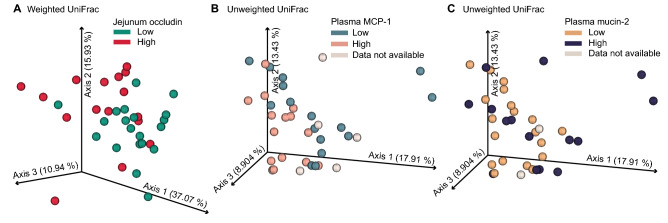
Changes in beta diversity of the cecal microbiome are associated with high and low expression of biological signatures of inflammation. **(A)** Principal coordinates ordination plot of weighted UniFrac coordinate analysis of the cecal microbiome was associated with low (pg/ml, pine green, *n* = 20) and high jejunum occludin concentrations (pg/ml, red, *n* = 16). Axis 1 explains 37.07%, axis 2 explains 15.93%, and axis 3 explains 10.94% of the variance in the microbiome data analysis such that a total of 62.94% of variance in the dataset is represented here. **(B)** Principal coordinates ordination plot of unweighted UniFrac analysis of the cecal microbiomes showing separation of samples with low plasma monocyte chemoattractant protein-1 concentrations (MCP-1, pg/ml) (blue-grey, *n* = 16) and high plasma MCP-1 concentrations (apricot, *n* = 14). Plasma MCP-1 data were not collected for *n* = 6 samples (grey). **(C)** Principal coordinates ordination plot of unweighted UniFrac analysis of the cecal microbiomes showing separation of samples with low (pg/ml, goldenrod, *n* = 19) and high plasma mucin-2 concentrations (pg/ml, deep purple, *n* = 15). Mucin-2 data were not collected for *n* = 2 samples (grey). **(B,C)** Principal coordinates ordination of unweighted UniFrac: axis 1 explains 17.91%, axis 2 explains 13.43%, and axis 3 explains 8.90% variance in microbiome analysis such that a total of 40.24% of the variance in the dataset is represented here. *MCP-1* monocyte chemoattractant protein-1.

However, composition of the cecal microbiome as assessed by weighted UniFrac was correlated with jejunum occludin levels in such a way that low concentrations of jejunum occludin clustered around axes 1 and 2 (*pseudo-F* = 4.22, *p* = 0.001; Fig. 4A). In addition, the composition of the cecal microbiome as assessed by unweighted UniFrac distance metric was associated with plasma MCP-1 concentrations, with a low plasma MCP-1 cluster around axis 2 and a high plasma MCP-1 cluster around axis 3 (*pseudo-F* = 1.87, *p* = 0.019; Fig. 4B). Finally, composition of the cecal microbiome as assessed by unweighted UniFrac distance metric was associated with plasma mucin-2 concentrations, with low mucin-2 concentrations associated with ordinal clustering around axes 2 and 3 (*pseudo-F* = 1.64, *p* = 0.043; Fig. 4C).

### Analysis of taxonomic differences in the cecal microbiome using ANCOM-II in relation to factors affecting alpha and beta diversity

**Figure 5:**
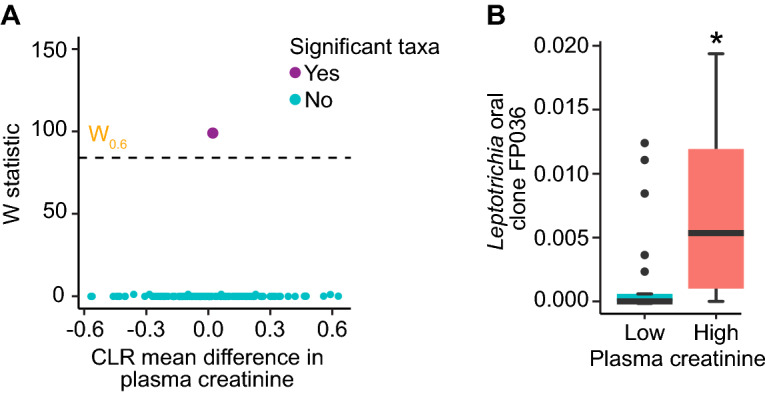
Several features in a linear mixed-effects analysis of composition of microbiomes (ANCOM-II)-based analysis of the cecal microbiome were used to identify taxa driving differences in plasma creatinine responses to polytrauma (PT) and hemorrhagic shock (HS). ANCOM-II volcano plots showing W-statistic against center-log ratio (CLR)-fold change differences between **(A)** low and high plasma creatinine levels, with non-significant taxa under the coefficient of concordance threshold of 0.6 (dashed-line) and significant taxa above the threshold. Structural zeros are points identified by ANCOM-II in peach; non-structural zeros, or taxa with non-infinity W-statistic values, are in blue. Relative abundances of **(B)** *Leptotrichia* oral clone FP036, indicated as a significant, non-structural zero taxon (*W*-statistic: 99) in panel **(A)**. The bottoms and tops of boxes indicate the first and third quartiles, respectively; whiskers indicate the 1.5 interquartile range beyond the upper and lower quartiles. Values outside the whiskers are indicated by black circles. *ANCOM-II* analysis of composition of microbiomes II, *CLR* center-log ratio, *HS* hemorrhagic shock, *PT* polytrauma*.*

Linear mixed-effects model analysis of the cecal microbiome using ANCOM-II identified one significant feature above the coefficient of concordance threshold of 0.6 that was differentially abundant in the low/high analysis of plasma creatinine (Fig. 5A). In the analysis of low and high plasma creatinine, *Leptotrichia* oral clone FP036 (*W*-statistic = 99; Fig. 5B) was the only differentially abundant feature detected by ANCOM-II; its relative abundance was significantly increased in the high plasma creatinine group.

### Supervised machine learning classification of samples based on microbial features

Random forest is a supervised machine learning algorithm that was used to evaluate sample classification of low and high inflammatory marker levels based on microbial features in this analysis. With sufficient computational ability and a relatively small dataset of *n* < 50, a nested cross-validation approach (*k* = fivefold) was employed to reduce bias and improve model performance^54^. Further unbiased classification of samples based on microbial composition allowed us to better understand how injury severity affected the cecal microbiomes of mice in this study beyond ANCOM-II-based analysis of taxa driving differences. Model performance and prediction accuracy were based on receiver operating characteristic (ROC) scores that evaluated predictive true positive rates (TPR) against false positive rates (FPR).

**Figure 6:**
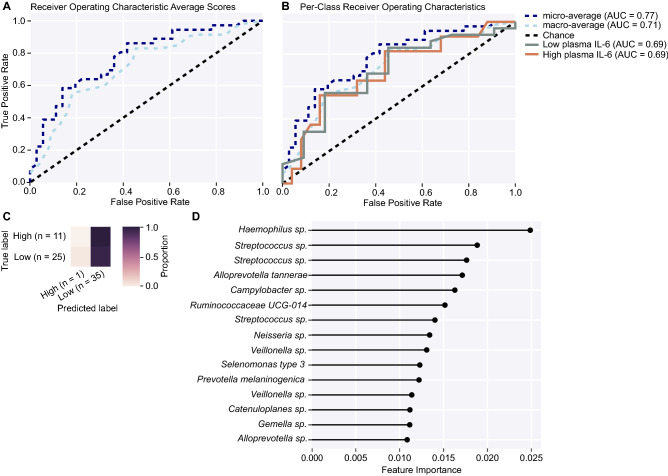
Machine learning classification accuracy in a nested cross-validation random forest model built to predict plasma IL-6 concentrations in mice from this study based on features of the cecal microbiome. Feature inputs from the raw feature table included in this analysis consisted of all bacteria matched to the Silva_132 ribosomal RNA gene reference database with 99% accuracy. **(A)** Receiver operating characteristic (ROC) plot shows macro-average precision (of each IL-6 classification equally-weighted, light blue dashed line, AUC = 0.71) at several true-positive rate (TPR) against false-positive rate (FPR) thresholds; micro-average precision (averaged metrics across each sample, dark blue dashed line, AUC = 0.77) at several TPR against FPR thresholds; and classification error rate achieved by random chance (black dashed line). **(B)** Per-class ROC shows the accuracy results for low (green) and high (orange) plasma IL-6 concentration classifications in this study. The overall accuracy in classifying low and high plasma IL-6 in mice was 69%. **(C)** The prediction matrix shows that a large proportion of samples were correctly classified in the low plasma IL-6 group. However, random forest classification performance was poor in utilizing features to identify samples in the high plasma IL-6 group; *n* = 10 samples were incorrectly classified in the low category for a total of *n* = 35. More accurate assessments of the True Label by the algorithm are correlated with darker shading. **(D)** Top 15 feature rankings in the random forest analysis. *AUC* area-under-the-curve, *FPR* false positive rate, *ROC* receiver operating characteristics, *TPR* true positive rate.

Of all the inflammatory markers that were associated with differences in alpha or beta diversity of the cecal microbiome, model performance as shown by area-under-the-curve (AUC) macro- and micro-average values for prediction of plasma IL-6 concentration based on features of the cecal microbiome were greater than would have been predicted by chance alone (AUC = 0.71 and 0.77, respectively; Fig. 6A). Per class ROC plots showed that the classification of samples to low or high plasma IL-6 concentration was 69% accurate (Fig. 6B). The confusion matrix for the plasma IL-6 concentration model demonstrated that samples were always correctly predicted for the low plasma IL-6 group and only 9% of the time for the high plasma IL-6 group (Fig. 6C). Inherent unevenness in the distribution of low and high true labels may have led to bias in classification error rates, as is the case with plasma IL-6 concentration where the data were split based on a threshold of 300 pg/ml^55,56^ rather than evenly-matched groups. The feature importance plot in Fig. 6D shows that many bacteria drive the classification of low and high plasma IL-6 levels, including *Alloprevotella* spp., *Campylobacter* spp., *Catenuloplanes* spp., *Gemella* spp., *Haemophilus* spp., and *Neisseria* spp. Additional features included *Alloprevotella tannerae, Prevotella melaninogenica, Ruminococcaceae* group UCG-014, *Selenomonas* type 3, and multiple features in the genus *Streptococcus* and *Veillonella* (Fig. 6D)*.*

**Figure 7:**
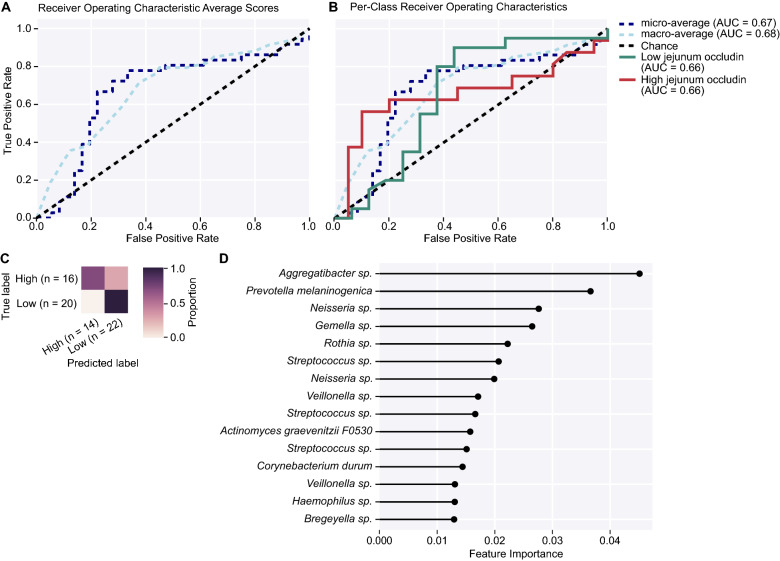
Machine learning classification accuracy in a nested cross-validation random forest model built to predict jejunum occludin concentrations (in pg/ml) based on features of the cecal microbiome in mice from this study. Feature inputs from the raw feature table included in this analysis consisted of all bacteria matched to the Silva_132 ribosomal RNA gene reference database with 99% accuracy. **(A)** Receiver operating characteristic (ROC) plot shows macro-average precision (each jejunum occludin classification equally-weighted, light blue dashed line, AUC = 0.68) at several true-positive rate (TPR) against false-positive rate (FPR) thresholds; micro-average precision (averaged metrics across each sample, dark blue dashed line, AUC = 0.67) at several TPR against FPR thresholds; and classification error rate achieved by random chance (black dashed line). **(B)** Per-class ROC shows the prediction results for low (forest green, AUC = 0.66) and high (red, AUC = 0.66) jejunum occludin classifications in study mice. The overall accuracy in classifying low and high jejunum occludin in study mice was 66%. **(C)** The prediction matrix shows that all but 2 samples were correctly classified by the machine learning algorithm. More accurate assessments of the true group designation by the algorithm are correlated with darker shading. **(D)** Top 15 feature rankings in the random forest analysis. *AUC* area-under-the-curve, *FPR* false positive rate, *ROC* receiver operating characteristics, *TPR* true positive rate.

The random forest predictions for jejunum occludin prevalence based on features of the cecal microbiome were also significant as indicated by the macro-average (AUC = 0.68) and micro-average (AUC = 0.67) values, which were both greater than chance alone (Fig. 7A). Per class ROC plots showed that the classification of samples to low or high jejunum occludin was 66% accurate (Fig. 7B). The confusion matrix shown in Fig. 7C indicates that the algorithm could correctly classify samples into the high jejunum occludin group, with an accuracy of 91% for the low jejunum occludin group. The feature importance plot in Fig. 7D demonstrates that many bacteria drive the classification of low and high jejunum occludin levels, including *Actinomyces graevenitzii* group F0530, *Aggregatibacter* spp., *Bregeyella* spp., *Corynebacterium durum*, *Gemella* spp., *Haemophilus* spp., *Prevotella melaninogenica*, and *Rothia* spp. There were also multiple features in the genus *Neisseria* spp., *Streptococcus* spp., and *Veillonella* spp. (Fig. 7D). It is important to note that the following features were observed as important drivers in both machine learning classification schemes of plasma IL-6 and jejunum occludin: *Neisseria* spp., multiple members of *Veillonella* spp., *Streptococcus* spp., *Haemophilus* spp., *Prevotella melaninogenica,* and *Gemella* spp.

## Discussion

In the present study, (1) we could not confirm the hypothesis that as early as 4 h after experimental PT + HS, the composition of the intestinal microbiome is significantly altered in comparison to respective sham-treated mice; more specifically, we did not find any changes in microbial alpha diversity or microbial community structure directly associated with types of injury according to experimental group allocation; however, (2) we did observe a general association of measures of intestinal microbial alpha and beta diversity with markers of systemic and local gut inflammation as well as intestinal barrier functioning. Thus, the data support the hypothesis that gut microbiome composition at the time of trauma affects both gut permeability and local/systemic inflammation.

Increases in systemic inflammation in this study were associated with alpha- and beta-diversity measures in the cecal microbiome. The combination of PT and HS led to broad increases in systemic inflammation that were not observed in any other group, including mice injured via PT or HS alone^5^. In the present analysis, the observed dramatic increases in systemic proinflammatory markers, such as plasma MCP-1 and IL-6, and markers of endothelial barrier damage, such as JAM-A, were associated with increases in alpha-diversity metrics of richness (Shannon’s diversity) and evenness (Pielou’s *J’* evenness). In addition, plasma MCP-1 concentration was associated with global changes in cecal microbial composition as measured by unweighted UniFrac distances. In this regard, the over-representation of PT + HS animals in the “high” groups of several of the assessed biological variables, and the separation of high plasma MCP-1 and high plasma mucin-2 concentrations based on unweighted UniFrac analysis points to a relationship between the extent of systemic inflammation and organ/barrier dysfunction markers and the community structure of the gut microbiome; this relationship may be partly dependent on the severity of the insult although we did not find significant differences between animals according to their experimental group allocation, presumably due to the analysis at a very early time point post-injury in a relatively small number of animals per group. Furthermore, as a limitation to the study, we observed a relatively high baseline diversity even in untreated control animals based on the study design which necessitated ordering mice from a commercial vendor in several cohorts over a longer time span which may cause significant differences in microbiome composition^57^. Nevertheless, we observed an increase in microbial richness dependent on the amount of perfused NE required for blood pressure maintenance, indicating that the extent of circulatory instability exerts a certain influence on the intestinal microbial composition, or that infusion of NE per se alters the gut microbiome. It should be noted that NE itself may alter the inflammatory response after injury^58^; whether higher systemic amounts of NE influence the microbiome directly (e.g., see Hughes et al.^59^) or indirectly via the well-described vasoconstriction of the gut vessels and thus potential gastrointestinal hypoxia remains unanswered.

The gut microbiome diversity and composition were also associated with inflammatory and gut permeability markers within both the jejunum and colon. Increases in alpha diversity were associated with increased IL-6 in both the jejunum and plasma measurements. The changes in cytokine secretion characterized by multiple inflammatory markers elevated in PT + HS indicates that inflammation in this preclinical model spreads from circulation into numerous organ systems. Plasma IL-6 in particular has been shown to increase tissue expression of the receptor for the complement activation product C5a in a model of septic shock^60^. Furthermore, intestinal epithelial cells are known to respond to C5a (which can induce all classical signs of inflammation) by increasing permeability of monolayers such as the gut mucosae^61^. Elevated generation of C5a in intestinal tissues thereby leads to impairments in gut-barrier function and integrity, increasing the likelihood of bacterial translocation via opsonization, microbial dysbiosis, and bacteremia, all of which possibly results in fatal sepsis^62,63^. In fact, C5 deficiency or C5a receptor inhibition protects mice against HS by significantly reducing intestinal damage severity^64^. Interestingly, direct antimicrobial activity of C3a, although not of C5a, has been demonstrated in vitro^65,66^ which in principle may contribute to direct alterations of the microbiome.

It is conceivable that immediate alterations in the gut microbiome after injury induce intestinal barrier dysfunction and thus allow bacterial translocation into the circulation, causing a strong systemic proinflammatory immune response prior to the onset of sepsis. Furthermore, preexisting individual variability in the gut microbiome at the time of trauma may have influenced the physiological response; for instance, a microbiome with a high relative abundance of LPS-containing gram negative bacteria might result in increased inflammation in response to trauma. A previous report proposed that a healthy gut microbiome reveals protective effects in the presence of gut inflammation and “leaky gut” through its ability to maintain tight junctions in the intestinal epithelium by regulating the expression of ZO-1 proteins^67^. Of note, intestinal ZO-1 expression was reduced in our PT + HS animals^6^, which is likely to be associated with a functional impairment of the gut barrier. In this regard, supervised machine-learning analysis using nested cross-validation random forest strategies suggested that various opportunistic pathogens are drivers of inflammation. The results demonstrate that animals within the low plasma IL-6 group regardless of treatment are more easily distinguished by microbial composition than animals with high IL-6, suggesting that greater levels of systemic inflammation are correlated with increased variability in microbial composition. The association of similar bacteria with differences in plasma IL-6 and jejunum occludin indicate that a relationship may exist between systemic inflammation and intestinal tight-junction integrity. Dephosphorylation of the tight-junction protein occludin and a loss of blood–brain barrier structure have been shown to correlate with the onset of inflammation in a murine model of multiple sclerosis (Morgan et al.^68^). This increase in barrier permeability, which can be induced by cytokines such as IL-6^69^ is likely to also occur in the jejunum.

Hemorrhagic shock may increase vulnerability to sepsis after severe PT, inducing an inflammatory cascade that ultimately results in multi-organ failure and death^26^. HS-induced blood loss and the subsequent halt in urine output by the kidneys allows the body to retain blood volume in the short term. However, this compensatory mechanism also results in the retention of metabolic waste byproducts (e.g., creatinine) and inflammatory signals (e.g., plasma cytokines) that would normally be filtered out during homeostasis. The retention of these factors contributes to increased risk of complications such as fatal sepsis. Interestingly, we found high systemic creatinine concentrations to be associated with increased alpha diversity, suggesting an interaction between the dysfunction of the primarily uninjured kidney and the intestine, presumably via the kidney-gut axis. The accumulation of plasma creatinine associated with changes in microbial diversity at the subject level (alpha diversity) and the observed association between the relative abundance of *Leptotrichia* oral clone FP036 and plasma creatinine concentrations might thereby be a byproduct of the renal feedback loop initiated by HS. While our data do not reveal direction of causality, we demonstrate a relationship between increases in the relative abundance of *Leptotrichia* and organ dysfunction, hinting at enhanced intestinal proliferation of opportunistic pathogens normally associated with the oral microbiome and increased susceptibility to organ failure and sepsis. Taken together, these findings are supported by several previous studies in models of severe injury leading to sepsis, demonstrating that critical illness correlates with significant alterations in the microbiome^70–73^.

Our restriction to linear correlation analyses may have prevented the detection of other, non-linear correlations between physiological parameters and those of microbial richness or evenness. Furthermore, a certain heterogeneity in microbial and inflammatory responses among individual mice is well-supported in the literature^74^. As a limitation of the study, we did not record blood gas tensions, acid–base status, nor lactate. Anesthesia-induced systemic respiratory acidosis especially in animals after TXT may have affected intestinal villous perfusion, mucosal integrity, and, hence, the gut flora. Also, we investigated an ultra-early time window for changes of the microbiome. Later time points such as 24 or 72 h after trauma should be addressed in future studies to get a temporal resolution of the microbiome alterations.

Previous studies have shown that severe injury causes an increase in richness and evenness of the gut microbiome but have neglected to elaborate on this observation^22,75^. We postulate that more pathogenic strains of bacteria that are present in low relative abundances prior to injury may find particular niches in a more hostile physiological environment following severe physical injury, thus increasing richness and evenness. For example, based on the employed machine-learning analysis of microbial features, our data suggest that specific microbial drivers of systemic inflammation and barrier dysfunction are members of the oral microbiome, including *Neisseria*, *Veillonella*, *Streptococcus*, *Haemophilus*, *Prevotella melaninogenica*, and *Gemella*^76^. Alternatively, it is possible that beneficial bacteria are decreased in the presence of homeostatic instability. In order to elucidate the mechanisms behind the observed increases in alpha diversity seen, future studies would require larger sample sizes.

In conclusion, our results demonstrate that systemic and local inflammation as well as intestinal barrier dysfunction are associated with the diversity and community composition of the cecal microbiome very early after trauma with an overabundance of oral pathogens. Since the type of insult determines the magnitude of systemic and local inflammatory responses as well as organ injury patterns, longitudinal studies in larger group sizes will elucidate the extent to which specific traumatic insults intercommunicate with the intestinal microbial composition and will shed light on the directionality of the effects observed in this study.

## Supplementary Information

Supplementary Tables.

## Abbreviations

ALI: Acute lung injury
ANCOM: Analysis of composition of microbiomes
ASV: Amplicon sequence variants
AUC: Area-under-the-curve
BAL: Bronchoalveolar lavage
BSTFA: *N,O-*Bis(trimethylsilyl)trifluoroacetamide
C3a: Complement component C3a
C5a: Complement component C5a
CLR: Center-log ratio
Ctrl: Non-treated control
DADA2: Divisive amplicon denoising algorithm 2
dsDNA: Double-stranded DNA
EDTA: Ethylenediaminetetraacetic acid
ELISA: Enzyme-linked immunosorbent assay
EMP: Earth microbiome project
FDR: False discovery rate
FPR: False positive rate
Hb: Blood hemoglobin
HS: Hemorrhagic shock
IBD: Inflammatory bowel disorder
IL-6: Interleukin 6
JAM-A: Junctional adhesion molecule A
MAP: Mean arterial pressure
MCP-1: Monocyte chemoattractant protein-1; C–C motif chemokine ligand 2 (CCL2)
MOF: Multiple organ failure
MUC2: Mucin 2
NE: Norepinephrine
PBS: Phosphate-buffered saline
PCoA: Principal coordinates analysis
PERMANOVA: Permutational analysis of variance
PT: Polytrauma
PT + HS: Polytrauma and hemorrhagic shock
QIIME2: Quantitative insights into microbial ecology 2
ROC: Receiver operating characteristics
SEPP: SaTé-enabled phylogenetic placement
Sham: Anesthetized and catheterized
TBI: Traumatic brain injury/closed head injury
TPR: True positive rate
TXT: Thoracic trauma/blunt bilateral chest trauma
V4: Hypervariable region 4
ZO-1: Zonula occludens protein 1
16S rRNA: 16 Small subunit ribosomal RNA
